# Computational Insights into the Potential of Withaferin-A, Withanone and Caffeic Acid Phenethyl Ester for Treatment of Aberrant-EGFR Driven Lung Cancers

**DOI:** 10.3390/biom11020160

**Published:** 2021-01-26

**Authors:** Vidhi Malik, Vipul Kumar, Sunil C. Kaul, Renu Wadhwa, Durai Sundar

**Affiliations:** 1 DBT-AIST International Laboratory for Advanced Biomedicine (DAILAB), Department of Biochemical Engineering & Biotechnology, Indian Institute of Technology (IIT) Delhi, Hauz Khas, New Delhi 110 016, India; vidhi0205@gmail.com (V.M.); vipul.kumar@dbeb.iitd.ac.in (V.K.); 2 AIST-INDIA DAILAB, DBT-AIST International Center for Translational & Environmental Research (DAICENTER), National Institute of Advanced Industrial Science & Technology (AIST), Tsukuba 305-8565, Japan; s-kaul@aist.go.jp

**Keywords:** EGFR, exon 20 insertion mutations, Withaferin A, Withanone, Caffeic Acid Phenethyl Ester, ATP competitive inhibitors, lung cancer, therapy

## Abstract

The anticancer activities of Withaferin-A (Wi-A) and Withanone (Wi-N) from Ashwagandha and Caffeic Acid Phenethyl Ester (CAPE) from honeybee propolis have been well documented. Here, we examined the binding potential of these natural compounds to inhibit the constitutive phosphorylation of epidermal growth factor receptors (EGFRs). Exon 20 insertion mutants of EGFR, which show resistance to various FDA approved drugs and are linked to poor prognosis of lung cancer patients, were the primary focus of this study. Apart from exon 20 insertion mutants, the potential of natural compounds to serve as ATP competitive inhibitors of wildtype protein and other common mutants of EGFR, namely L858R and exon19del, were also examined. The potential of natural compounds was compared to the positive controls such as erlotinib, TAS6417 and poziotinib. Similar to known inhibitors, Wi-A and Wi-N could displace and binds at the ATP orthosteric site of exon19del, L858R and exon20, while CAPE was limited to wildtype EGFR and exon 20 insertion mutants only. Moreover, the binding free energy of the natural drugs against EGFRs was also comparable to the positive controls. This computational study suggests that Wi-A and Wi-N have potential against multiple mutated EGFRs, warranting further *in vitro* and *in vivo* experiments.

## 1. Introduction

Lung cancer is a leading cause of death worldwide [1]. It is primarily categorized into two subtypes-small cell lung cancer (SCLC) that accounts for 15% of the lung cancer cases and non-small cell lung cancer (NSCLC) that accounts for the remaining 85% [2,3,4,5]. The most common cause of NSCLC reported in several studies is the constitutive activation of EGFR (Epidermal Growth Factor Receptor) [6,7]. EGFR is a 170-kD transmembrane glycoprotein that promotes cell proliferation. It has an extracellular domain for ligand binding, a transmembrane (TM) region and intracellular tyrosine kinase and regulatory domains [8,9]. The binding of the EGFR receptor to its ligand (EGF) causes its homo-dimerization that stimulates its intrinsic tyrosine kinase activity, causing autophosphorylation of the receptor and phosphorylation of various substrates in the cell [10]. EGFR interacts with several signal transducing proteins, including protein kinase B (AKT/PKB) and mitogen-activated protein kinases (MAPK) that help in the transmission of growth and differentiation signals [11,12]. The vast majority of activating mutations in the EGFR gene have been reported as key-drivers in NSCLC. Among these, the single point mutation L858R in exon 21 and variable deletions of at least three amino acid residues in exon 19 account for almost 85% of NSCLC cases. These classical activating mutants have diminished ATP binding ability and increased binding affinity for first-generation EGFR inhibitors over EGFR wildtype [7,13]. However, another set of EGFR gene mutation that is caused due to in-frame base pair insertions in exon 20 has been detected at a low frequency. These exon 20 insertion mutations have been commonly reported in the non-smoking Asian female population and people with adenocarcinoma histology, and they are known to cause constitutive activation of the EGFR and resistance to its inhibitors (gefitinib/erlotinib) [13,14]. NSCLC patients having exon 20 insertion mutations of EGFR show low response rate of 3–8% to first-generation inhibitors (gefitinib/erlotinib) and 8.7% to second-generation inhibitor afatinib [13].

In many previously reported clinical trials, it was found that the treatment with multiple EGFR inhibitor drugs could increase the overall survival rate of patients suffering from NSCLC. Until now, three generation of EGFR inhibitor drugs are available, which are approved by food and drug administration. The erlotinib and gefitinib are the reversible ATP competitive first-generation EGFR tyrosine inhibitor drugs, which were found to be effective in severe NSCLC with almost 72% response rate [13]. However, continuous treatment with these drugs leads to resistance within 12 months, due to accumulation of mutations [15]. The second-generation drugs, dacomitinib and afatinib, are irreversible inhibitors of EGFR that covalently interact with C797 residue and have been demonstrated to be effective against classical EGFR mutations. Treatment with dacomitinib and afatinib showed progression free survival benefit of 14.7 and >10 months, respectively, in patients with classical EGFR mutations. However, second-generation EGFR inhibitors have not found to be effective against exon 20 insertion mutant of EGFR [16]. The third-generation EGFR inhibitors, rociletinib and osimertinib, were designed to deal with emergence of T790M mutation as a mechanism of resistance to first-generation EGFR inhibitors. These inhibitors covalently bind to C797 and can inhibit T790M mutants as well. Among the third-generation inhibitors, osimertinib has been approved as second-line therapy for patients having T790M secondary mutations after treatment with erlotinib/gefitinib with an impressive response rate of more than 60% [13]. In addition, it has been approved for first-line therapy for classical EGFR mutations with median progression free survival benefit of 17.2 months, compared to 8.5 months for first-generation reversible inhibitors [17]. However, osimertinib also becomes ineffective within a period of one year due to C797S mutation in the EGFR of NSCLC patients [18]. Now, research is ongoing towards the development of novel and potent fourth-generation inhibitors of EGFR tyrosine kinases to deal with C797S mutation. The effect of osimertinib on exon 20 insertion mutants is still not clear; pre-clinical studies using patient-derived xenograft models and CRISPER-Cas 9 engineered cell lines showed better and selective inhibitory effect of osimertinib on D770_N771^InsSVD^ and V769_D770^InsASV^ as compared to afatinib [19]. A phase II clinical trial to assess inhibitory effect of osimertinib on patients harboring exon 20 insertion mutations is ongoing [13]. Poziotinib is another major clinical candidate that targets exon 20 insertion mutations, showing a preliminary response rate of 64% in a small cohort of patient in an ongoing phase II clinical trial, in contrast to <8% and 8.7% observed for erlotinib and afatinib, respectively [13]. Poziotinib is an irreversible inhibitor that interacts covalently with Cys797 residue. Poziotinib showed disappointing results in initial phase II clinical trial with NSCLC patients having classical EGFR mutations that acquired resistance to EGFR inhibitors via accumulation of secondary mutations such as T790M [20]. However, it showed promising inhibitory activity for exon 20 insertion mutations of EGFR in genetically engineered *in vitro* and *in vivo* models and in patient-derived xenograft models [21]. Due to its flexible and compact structure, it does not experience steric hindrances, unlike second- and third-generation EGFR inhibitors and can fit well in the small binding pocket of exon 20 insertion mutants of EGFR [21]. The action of poziotinib is not selective to EGFR exon 20 insertion mutations and targets wildtype EGFR as well, which may impose dose limitation in clinical settings [13]. TAS6417 is another irreversible inhibitor that is specifically designed to target ATP binding site of EGFR exon 20 insertion mutations and form covalent bond with Cys797 residue. It selectively targets exon 20 insertion mutations over wildtype EGFR and showed inhibition of EGFR phosphorylation and cell growth in genetically engineered *in vitro* and *in vivo* models [22].

Cancer cells are known to acquire resistance to synthetic drugs, and hence multidrug combination therapy is favored over single drugs. The main drawback of using multiple synthetic drugs against cancer is excessive toxicity [23] and has hence necessitated the search for natural drugs with preventive and therapeutic potential for cancer treatment. Here, we investigated the potentials and mechanisms of action of Ashwagandha derived two steroidal lactone bioactive withanolides, Withanone (Wi-N) and Withaferin-A (Wi-A), for treatment of aberrant EGFR-derived lung cancers. The Ashwagandha plant is known for its therapeutic properties in Ayurveda for the past 3000 years and has been an indispensable part of the Indian traditional home-medicine system [24,25]. Scientifically known as *Withania somnifera*, it possesses aphrodisiac, adaptogenic, rejuvenative, anti-inflammatory and anticancer properties [26] that have been assigned to its secondary metabolites, e.g., alkaloids, steroidal lactones and saponins. Wi-N and Wi-A have been reported to have anticancer activities that works through the inhibition of Mortalin-p53 interactions leading to activation of tumor suppressor activities of p53, downregulation of NFkB leading to inhibition of inflammation signaling, inhibition of MRN complex leading to accumulation of DNA damage and growth arrest of cancer cells and inhibition of VEGF and MMPs leading to inactivation of cancer cell migration and metastasis [27,28,29,30,31,32,33,34,35,36]. It has also been reported through cell culture assays that Wi-A induced apoptosis in NSCLC having both wildtype and mutant forms of EGFR [37]. In addition, Wi-A in combination with glucose metabolism targeted therapy was able to inhibit gefitinib resistant lung cancer cell lines [38]. Similarly, Caffeic Acid Phenethyl Ester (CAPE), a bioactive component from honeybee propolis, has been shown to possess anticancer and anti-metastasis activities through various mechanisms [34,39,40,41,42,43,44]. Several *in vitro* and *in vivo* based studies showed that CAPE and its derivatives can inhibit EGFR signaling pathway through downregulation of expression level of EGFR and phosphorylated EGFR proteins [45,46].

**Figure 1:**
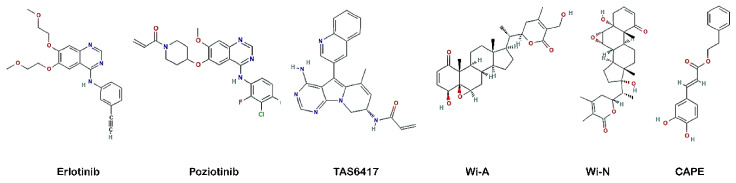
Structure of compounds selected for the study.

In this study, through an *in silico* approach, we investigated the potential of Wi-A, Wi-N and CAPE as aberrant EGFR inhibitors by comparing their binding to EGFR with respect to its known inhibitors, including erlotinib, TAS6417 and poziotinib (Figure 1). The ATP binding site of EGFR wildtype, exon 19 deletion mutant (exon19del), exon 21 (L858R) and exon 20 insertion mutants (D770_N771^InsNPG^, D770_N771^InsSVD^, V769_D770^InsASV^ and H773_V774^InsH^) were targeted to check the inhibitory effects of Wi-A, Wi-N and CAPE. erlotinib was taken as a negative control (as most exon 20 insertion mutants of EGFR are resistant to erlotinib). TAS6417 and poziotinib were taken as positive controls for their reported activity against exon 20 insertion mutations of EGFR [13,47]. We provide computational evidence that Wi-A, Wi-N and CAPE could inhibit ATP binding and autophosphorylation for aberrant EGFR.

## 2. Materials and Methods 

### 2.1. Structure Preparation of Proteins and Ligands

The initial crystal structure of Chain A of wildtype active conformation of EGFR complexed with ATP was retrieved from Protein Data Bank (PDB) with PDB ID: 2ITX [48]. The structure was modified by adding another chain of the same residues to form EGFR homodimer by aligning it with crystallized structure of EGFR dimer (PDB Id: 4LRM) using PyMOL [49]. The initial protein structure was prepared and processed by the addition of hydrogen atoms, filling missing side chains and minimization of structure using the OPLS3e force field with the protein preparation wizard of Schrodinger software [50]. Six mutants and a wildtype EGFR were chosen for the study. Out of the six mutants, one was exon19del, in which there were in-frame deletions of exon 19. This ranged from 729 to 761 amino acids where any frame within the given range could be deleted. The most frequently reported exon19del mutant, E746–A750, was selected for the study. The second mutant was exon 21 point mutation, in which any amino acid ranging from 824 to 875 could be mutated; for this study, L858R was chosen as it was most frequent [51]. The structures of these mutants were created by introducing mutations in wildtype EGFR–ATP complex followed by molecular dynamic (MD) simulation. The rest of the mutants were of exon 20 insertion mutations: D770_N771^InsNPG^ (PDB Id: 4LRM), D770_N771^InsSVD^, V769_D770^InsASV^ and H773_V774^InsH^. The structures of other EGFR exon 20 insertion mutants were created by introduction of mutations in EGFR D770_N771^InsNPG^ structure (PDB Id: 4LRM) using PyMOL followed by MD simulations to obtain modified conformation of proteins from stable simulation trajectories. These six mutants and a wildtype EGFR were targeted to check the inhibitory effects of natural compounds by molecular docking and MD simulations studies. For wildtype EGFR, exon19del and L858R mutants, erlotinib was taken as the positive control. For the study of exon 20 insertion mutants, erlotinib was taken as negative control and TAS6417 and poziotinib were taken as positive controls. The structures of erlotinib, TAS6417, poziotinib, CAPE, Wi-A and Wi-N were obtained from the PubChem database having IDs 176870, 117918742, 25127713, 5281787, 265237 and 21679027 respectively [52]. These ligand structures were prepared and processed for docking using the LigPrep tool of the Schrodinger suite [53].

### 2.2. Molecular Docking and MD Simulations to Check the Potential of Natural Compounds to Serve as ATP Competitive Inhibitors of EGFR Mutants

After processing and preparation of structures of all the protein and ligands, the Glide module of Schrodinger was used for performing molecular docking [53]. For docking studies, firstly, a grid box of 10 Å^3^ was generated by taking the centroid of the ATP binding residues of EGFRs using receptor grid generation tool. Further, the prepared ligands were docked at the binding site choosing the generated grid using glide Extra precision (XP) docking algorithm with flexible ligand sampling option, while keeping all other options as default [53,54]. 

Molecular docking of proteins with that of ligands was followed by MD simulations to check the stability of ligand at ATP binding site of the protein and monitor any conformational changes induced in protein–ligand complexes. The MD simulation of the prepared mutants and protein-ligand complexes were done using Desmond MD tool integrated with Maestro Schrodinger software. Each system was solvated with the TIP3P water model in an orthorhombic periodic boundary box. To prevent interaction of the protein complex with its own periodic image, the distance between the complex and the box wall was kept 10 Å. The system was then neutralized by the addition of appropriate number of Na^+^/Cl^−^ ions depending on the protein–ligand complex using OPLS3e forcefield. Energy of the prepared systems was minimized for 5000 steps using the steepest descent method or until a gradient threshold of 25 kcal/mol/Å was achieved [55,56]. It was followed by L-BFGS (Low-memory Broyden–Fletcher–Goldfarb Shanno quasi-Newtonian minimizer) until a convergence threshold of 1 kcal/mol/Å was met. Further, the minimized systems were equilibrated in seven steps in NVT and NPT ensembles using relax model system before simulation option in Desmond Schrodinger suite. The equilibrated systems were then subjected to 50–150 ns unrestrained MD simulations in NPT ensemble with 300 K temperature maintained by Nose–Hoover chain thermostat, constant pressure of 1 atm maintained by Martyna–Tobias–Kelin barostat and an integration time step of 2 fs with recording interval of 20 ps [53]. The competition at ATP binding site was further studied by docking of ATP at its binding site of all simulated protein–ligand complexes, and the protein–ligand–ATP complexes were simulated for another 50 ns. As the number of protein–ligand complexes to be simulated was quite high (approximately 80 systems), one protein–ligand system (EGFR D770_N771^InsSVD^–poziotinib complex) was simulated for 500 ns to estimate the simulation duration required to attain stability of ligand bound protein complexes. The RMSDs of protein and ligand in EGFR D770_N771^InsSVD^–poziotinib complex clearly showed that the protein–ligand complex had attained stability within first 50 ns of simulation (Figure S1A). The average representative structure of EGFR D770_N771^InsSVD^–poziotinib complex was attained from stable simulation trajectories of 50 and 500 ns, respectively, and superimposed. It was observed that ligands were stably interacting at the same site in both complexes (Figure S1B). The study of EGFR exon 20 insertion mutant–ligand complex for 500 ns simulation provided clear understanding of the behavior of the system subjected to MD simulation and helped in determining the duration of simulation required to attain stability of the system. Hence, all the protein–ligand and protein–ligand–ATP complexes were simulated for a time duration of 50–150 ns based on RMSD pattern of their simulation trajectories.

### 2.3. Analysis of MD Simulated Systems

Root Mean Square Deviation and Hydrogen bond profiling of each system was done using Visual Molecular Dynamics (VMD) version 1.9.4 [57]. The binding pocket volume of EGFR exon 20 insertion mutant structures was calculated using SiteMap module of Schrodinger suite. The superimposition of every simulated system was done with the wildtype structure to investigate the changes in structures using PyMOL. Specifically, changes in Activation loop (719–723), P loop (855–876) and the shift in αC-helix of each EGFR structure were investigated very carefully, and changes in the distance between K745 and E762 are also reported.

The MM/GBSA (molecular mechanics energies combined with the generalized Born and surface area continuum solvation) free binding energy was calculated using the prime module of the Schrodinger suite. The details of MM/GBSA binding free energy study using prime Schrodinger module is reported in [58]. Average structures from each trajectory were used for this computation using the following equations:

MM/GBSA ΔG_bind_ = ΔG_complex_ − (Δ G_receptor_ + Δ G_ligand_)

ΔG = ΔE_gas_ + ΔG_sol_ − TΔS_gas_

ΔE_gas =_ ΔE_int_ + ΔE_elec_ +ΔE_vdw_

ΔG_sol_ = ΔG_gb_ + ΔG_surf_

The prime module was used to compute all the energy component using the coordinates of complex, receptor and ligand using OPL3e forcefield. The binding free energy (ΔG_bind_) was dissociated into the binding free energy of the complex, receptor and ligand. The gas-phase interaction energy (ΔE_gas_) was calculated as the sum of electrostatic (ΔE_elec_) and vander waal (ΔE_vdw_) interaction energies, while internal energy was neglected. The solvation free energy (ΔG_sol_) contains non-polar (ΔG_surf_) and polar solvation energy (ΔG_gb_), which was calculated using the VSGB solvation model and OPL3e force field, while the entropy term was neglected by default.

## 3. Results

### 3.1. Computational Modeling of Exon 20 Insertion Mutants of EGFR

**Figure 2:**
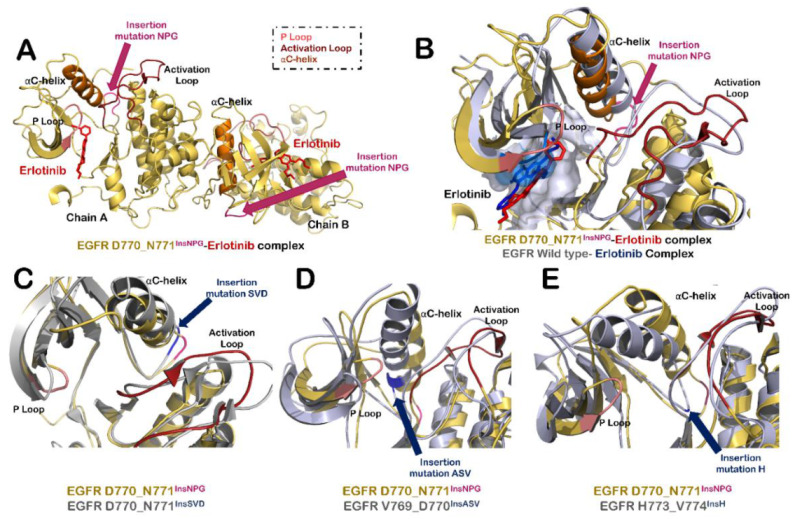
Structural modifications in EGFR protein due to insertion mutations in exon 20. (**A**) Structure of EGFR dimer having D770_N771^InsNPG^ mutation in complex with erlotinib. Insertion mutation is highlighted in pink color. Other highlighted structural elements around ATP binding site of protein include activation loop (shown in firebrick red color), α-C helix (orange) and P loop (salmon). (**B**) Superimposed structure of ATP binding site of Chain A of wildtype EGFR- erlotinib complex (grey-blue) with that of EGFR D770_N771^InsNPG^–erlotinib complex (yellow-red). (**C**) Superimposed structure of ATP binding site of Chain A of EGFR D770_N771^InsNPG^ (yellow-red) mutant with that of EGFR D770_N771^InsSVD^ (grey-blue). (**D**) Superimposition of ATP binding site of Chain A of EGFR D770_N771^InsNPG^ (yellow-red) with EGFR V769_D770^InsASV^ (grey-blue). (**E**) Superimposed structure of ATP binding site of Chain A of EGFR D770_N771^InsNPG^ (yellow-red) with that of EGFR H773_V774^InsH^ (grey-blue).

The structures of different exon 20 insertion mutants of EGFR were modeled by introducing mutations in EGFR D770_N771^InsNPG^ structure (PDB Id: 4LRM), followed by MD simulation until stability was attained in the RMSDs of simulation trajectories (Figure S2A). Exon 20 insertion mutations usually occur at C terminal end of αC-helix, which affect its orientation (Figure 2A). The αC-helix orientation plays a regulatory role in EGFR activation mechanism by pivoting from an outward (inactive state) to inward direction (active state) (Figure S3). The three residues insertion occurs at a pivot point that form a tight turn and might sterically inhibit the outward movement of αC-helix, thereby keeping the mutated structure in an active state [59] (Figure 2A,B). In addition, in the crystal structure of EGFR D770_N771^InsNPG^, EGFR was found to be in asymmetric dimer units with the same orientation as observed in the case of active EGFR dimer [59]. In the structure of EGFR D770_N771^InsNPG^–erlotinib complex, compared to that of the wildtype EGFR–erlotinib complex, a slight inward shift of αC-helix was observed (Figure 2B), which led to a smaller ATP binding pocket volume of 313.15 Å^3^ in the case of Chain A of EGFR D770_N771^InsNPG^–erlotinib complex as compared to 418.17 Å^3^ of the wildtype EGFR–erlotinib complex. 

The structure of other mutants of EGFR, namely D770_N771^InsSVD^, V769_D770^InsASV^ and H773_V774^InsH^, were modeled and compared with D770_N771^InsNPG^ to analyze differences in conformation among exon 20 insertion mutants of EGFR (Figure 2C–E). In the case of D770_N771^InsSVD^ mutation, no major differences were observed in the structures and it resembled the active conformation of D770_N771^InsNPG^ complex with inward orientation of αC-helix and extended activation loop segment (Figure 2C). However, in the case of V769_D770^InsASV^ insertion mutation of EGFR, extra helical turn was introduced in αC-helix, and the helix was also pushed slightly inward as compared to D770_N771^InsNPG^ structure (Figure 2D). Similarly, the insertion of histidine residue at position 773 in H773_V774^InsH^ mutant of EGFR adds rigidity to the movement of αC-helix and pushed it further in the inward direction (Figure 2E). In addition to this, the structure of this mutant was found to be in active state similar to the other exon 20 insertion mutants studied here.

### 3.2. Structural Differences between the Active and Inactive Conformation of EGFR Protein

The ATP binding domain of EGFR was targeted to investigate whether Wi-A, Wi-N and CAPE could bind and induce conformation changes in its structure. The active (PDB Id: 2ITX) and inactive conformations (PDB Id: 2GS7) [60] of the EGFR–ATP complex were examined by MD simulations. It was observed that, in the active conformation of EGFR–ATP complex (monomer), the distance between Lys745 and Glu762 was 5.7 Å, which was increased to 15.2 Å in the inactive conformation. This increase in distance caused an outward shift of αC-helix in the inactive state. Other differences observed in these two states were downward shift of P-loop region and helical turn formation in the activation loop of EGFR–ATP complex in the inactive conformation (Figure S3). Different structural aspects of EGFR were investigated in all docked complexes of EGFR mutants in complex with our control (erlotinib, poziotinib and TAS6417) and test (Wi-A, Wi-N and CAPE) inhibitors, such as distance between Lys745 and Glu762, the conformation of Activation loop and P loop, αC-helix and DFG motif orientation and hydrogen bond profile in both chains of EGFR [61]. 

### 3.3. Wi-A, Wi-N and CAPE Showed Potential as Competitive Inhibitors of ATP for EGFR Mutants

The binding ability of erlotinib, poziotinib, TAS6417, CAPE, Wi-A and Wi-N at ATP binding site of exon 20 insertion mutants (D770_N771^InsNPG^, D770_N771^InsSVD^, V769_D7701^InsASV^ and H773_V774^InsH^) were analyzed using molecular docking and MD simulations. It was observed that all compounds were able to bind at the ATP binding site of EGFR mutants with strong binding affinity. The protein RMSDs of all exon 20 insertion mutant–ligand complexes were also stable and comparable among all control and tested compounds except for the case of D770_N771^InsSVD^–CAPE complex (Figure S2B–E). The D770_N771^InsSVD^–CAPE complex attained stability at RMSD values of 5–6.5 Å in comparison to RMSDs of 3–4.7 Å to achieve stability for other D770_N771^InsSVD^–ligand complexes (Figure S2B). It was hence predicted that they could serve as ATP competitive inhibitors for all exon 20 insertion mutants (Table 1). In-depth analyses of various structural aspects of ATP binding site in EGFR exon 20 insertion mutant–inhibitor complexes revealed that all inhibitors compete with ATP at its binding site and maintain the protein in an active conformation. The active states of the protein of all mutant–inhibitor complexes were retained by maintaining the extended conformation of activation loop, DFG-*in* and αC-helix-*in* orientation of DFG motif and αC-helix, and the distance between Lys745 and Glu762 was not increased enough in any complex to flip αC-helix in an outward direction (Table 1 and Figure S4). To perceive the competition at the ATP binding site of mutants, the ATP molecule was also added to all EGFR exon 20 insertion mutant–inhibitor complexes. On addition of ATP at the binding site, competition among inhibitor and ATP was monitored to check whether the inhibitor could be displaced from its site by ATP binding in all EGFR mutant–inhibitor–ATP complexes. Of note, it was observed that most of our natural compounds were able to compete with ATP similarly to our positive controls (TAS6417 and poziotinib) with the exceptions of one mutant for each inhibitor. The detailed analyses of interactions of Wi-A, Wi-N and CAPE and the control inhibitors at ATP binding site of EGFR mutants in the presence and absence of ATP are summarized in Table 1.

### 3.4. All Compounds Showed Potential as ATP Competitive Inhibitors for EGFR Exon 20 Insertion Mutants (D770_N771^InsSVD^ and V769_D770^InsASV^)

**Figure 3:**
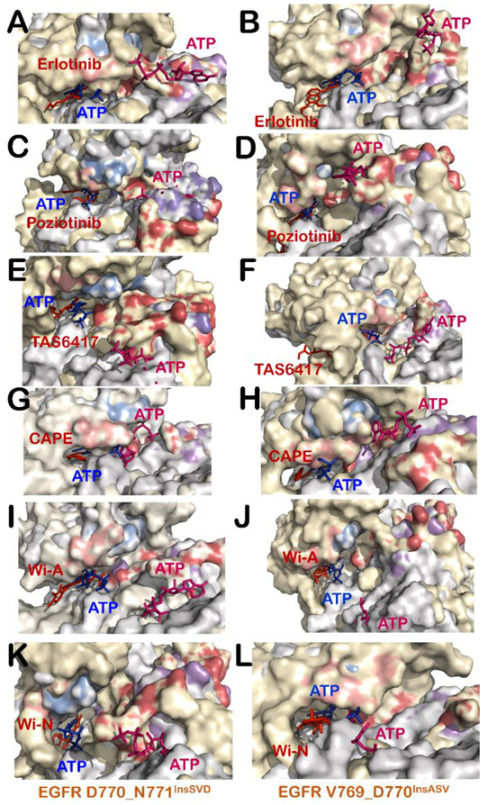
Superimposition of wildtype EGFR–ATP (grey-blue) complex with D770_N771^InsSVD^-inhibitor–ATP (yellow-red-pink) complexes (**A**,**C**,**E**,**G**,**I**,**K**) and V769_D770^InsASV^-inhibitor–ATP (yellow-red-pink) complexes (**B**,**D**,**F**,**H**,**J**,**L**) showing interaction of ATP and inhibitors at the ATP binding site: erlotinib (**A**,**B**); poziotinib (**C**,**D**); TAS6417 (**E**,**F**); CAPE (**G**,**H**); Wi-A (**I**,**J**); and Wi-N (**K**,**L**). P Loop and activation segment of wildtype EGFR–ATP complex is shown in marine blue and purple color and that of EGFR exon 20 insertion mutant–inhibitors–ATP complexes is shown in salmon and firebrick red color.

The ability of control and test compounds to restrict binding of ATP at its binding site was monitored in all EGFR exon 20 insertion mutant–inhibitor complexes. It was observed that, in the case of D770_N771^InsSVD^ and V769_D770^InsASV^ mutants, all control (Figure S4G–I,M–O) and test compounds (Figure S4J–L,P–R) were able to maintain the active state of mutant proteins and showed stable binding at ATP binding site. In addition, ATP was displaced to a distant site in all EGFR exon 20 insertion mutant–inhibitor–ATP complexes (Figure 3). The study of interactions formed by inhibitor and ATP in D770_N771^InsSVD^-inhibitor–ATP complexes and V769_D770^InsASV^-inhibitor–ATP complexes also revealed that ATP was unable to replace inhibitors, including erlotinib, from ATP binding site and formed interactions with distant residues (Tables S1–S6). Overall, the results indicate that both the control (erlotinib, poziotinib and TAS6417) and the test (Wi-A, Wi-N and CAPE) compounds could serve as ATP competitive inhibitors for EGFR exon 20 insertion mutants, D770_N771^InsSVD^ and V769_D770^InsASV^.

### 3.5. TAS6417 Could Serve as ATP Competitive Inhibitor of All the Four Exon 20 Insertion Mutants

**Figure 4:**
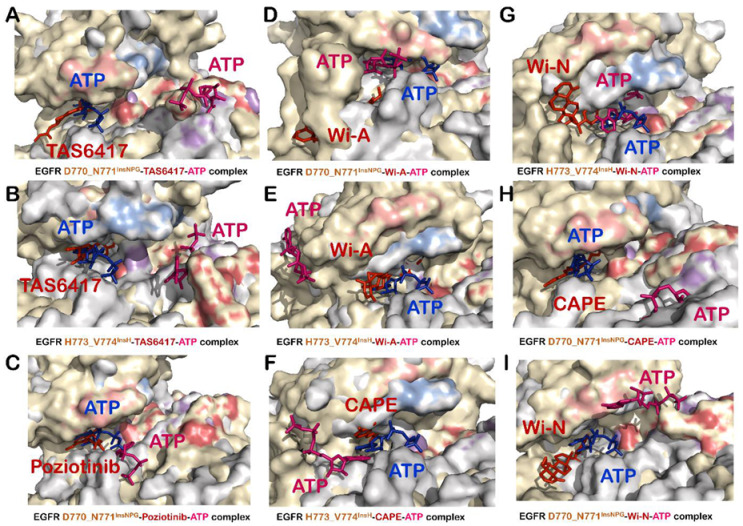
Superimposition of wildtype EGFR–ATP (grey-blue) complex with EGFR exon 20 insertion mutant–inhibitor–ATP (yellow-red-pink) complexes to unveil competition of inhibitors with ATP at ATP binding site of: (**A**) D770_N771^InsNPG^–TAS6417–ATP complex; (**B**) H773_V774^InsH^–TAS6417–ATP complex; (**C**) D770_N771^InsNPG^–poziotinib–ATP complex; (**D**) D770_N771^InsNPG^–Wi-A–ATP complex; (**E**) H773_V774^InsH^–Wi-A–ATP complex; (**F**) H773_V774^InsH^–CAPE–ATP complex; (**G**) H773_V774^InsH^–Wi-N–ATP complex; (**H**) D770_N771^InsNPG^–CAPE–ATP complex; and (**I**) D770_N771^InsNPG^–Wi-N–ATP complex. P Loop and activation segment of wildtype EGFR–ATP complex is shown in marine blue and purple color and that of EGFR exon 20 insertion mutant–inhibitors–ATP complexes is shown in salmon and firebrick red color.

TAS6417 showed good binding affinity at the ATP binding site of all EGFR exon 20 insertion mutants (Table 1). A slight increase in distance between Lys745 and Glu762 was observed in the case of D770_N771^InsSVD^ and H773_V774^InsH^ mutants due to interaction of TAS6417 at ATP binding site (Table 1 and Figure S4I,U). However, it failed to flip the αC-helix orientation to outward (Table 1 and Figure S4I,U). The downward shift of the P-loop was observed in the case of V769_D770^InsASV^ only (Figure S4O). DFG-*in* conformation was maintained in all EGFR exon 20 insertion mutant–TAS6417 complexes (Figure S4C,I,O,U). Hence, it can be suggested that TAS6417 could act as an ATP competitive inhibitor and maintain the active conformation of EGFR exon 20 insertion mutants. Subsequently, the competition at the ATP binding site was examined by the addition of ATP in EGFR exon 20 insertion mutant–TAS6417 complexes. The ATP was not able to replace TAS6417 from any mutant and displaced to a site distant from the ATP binding site (Figure 3E,F and Figure 4A,B). The investigations on interactions formed by TAS6417 and ATP in EGFR exon 20 insertion mutant–TAS6417–ATP-complexes also showed that TAS6417 was able to interact with the majority of ATP interacting residues in all complexes, whereas ATP was shifted to another site (Table S3).

### 3.6. Poziotinib and Wi-A Could Not Serve as ATP Competitive Inhibitors for D770_N771^InsNPG^ Mutant, Whereas CAPE and Wi-N Could Not Inhibit H773_V774^InsH^ Mutant of EGFR

The computational analyses of competition between the test compounds and ATP at the ATP binding site revealed some of the exceptions of the effectiveness of each compound for EGFR exon 20 insertion mutants. In the case of D770_N771^InsNPG^, poziotinib and Wi-A failed to inhibit the interaction of ATP with its interacting residues at the ATP binding site. D770_N771^InsNPG^–poziotinib–ATP complex showed binding of ATP in the correct orientation at a site very close to its real binding site taken by poziotinib (Figure 4C and Table S2) that showed stable binding with binding energy −59.5 Kcal/mol at Chain A and −70.14 Kcal/mol at Chain B. Of note, the binding energies of poziotinib were much stronger than those of ATP (−20.56 Kcal/mol at Chain A and −25.61 Kcal/mol at Chain B) in D770_N771^InsNPG^–poziotinib–ATP complex. Apart from D770_N771^InsNPG^, poziotinib formed stable interactions at the ATP binding site and successfully inhibited interactions of ATP with the ATP binding site of D770_N771^InsSVD^ (Figure 3C), V769_D770^InsASV^ (Figure 3D) and H773_V774^InsH^ (Table S2) mutants. However, in the case of D770_N771^InsNPG^–Wi-A–ATP complex, Wi-A was unable to bind at the ATP binding site and hence ATP was able to bind at its site (Figure 4D). The analyses of interactions of Wi-A and ATP in EGFR exon 20 insertion mutants showed that Wi-A was able to inhibit the interaction of ATP at its binding site for D770_N771^InsSVD^ (Figure 3I), V769_D770^InsASV^ (Figure 3J) and H773_V774^InsH^ (Figure 4E) complexes except for the case of D770_N771^InsNPG^ (Figure 4D), where ATP was able to form all its interactions (Table S5). Similarly, for H773_V774^InsH^ mutant, CAPE and Wi-N could not restrict binding of ATP near its site and ATP was able to form interactions with some of the ATP interacting residues (Figure 4F,G and Tables S4 and S6). In addition, the binding energy of ATP was comparable to those of CAPE and Wi-N in H773_V774^InsH^–CAPE–ATP complex and H773_V774^InsH^–Wi-N–ATP complex, respectively (Table 1). With the exception of H773_V774^InsH^ mutant, both CAPE and Wi-N were able to successfully inhibit binding of ATP at its binding site for D770_N771^InsSVD^ (Figure 3G,K), V769_D770^InsASV^ (Figure 3H,L) and D770_N771^InsNPG^ mutants (Figure 4H,I and Tables S4 and S6).

### 3.7. Combination of Wi-A and Wi-N Could Target Activity of All Four Exon 20 Insertion Mutants, L858R and Exon19del Mutants as Well as Wildtype EGFR

The interactions of erlotinib and the natural compounds with L858R mutant and exon19del mutant were also examined. We found that, in all the L858R–inhibitor complexes, the distance between Lys745 and Glu762 was not increased and αC-helix orientation remains inward (Table 2). Hence, in all these complexes, L858R mutants seemed to be in an active state. Although the docking score of Wi-N (at Chain A −4.05 Kcal/mol; at Chain B −3.44 Kcal/mol) and Wi-A (at Chain A −3.73 Kcal/mol; at Chain B −5.99 Kcal/mol) was slightly less than the erlotinib (at Chain A −7.38 Kcal/mol; at Chain B −7.38 Kcal/mol), both Wi-A and Wi-N were stable throughout the 150 ns of the MD simulations. Met793 was a common residue, which was making the hydrogen bond in most of the complexes. Wi-A or Wi-N bound L858R mutant could efficiently inhibit the binding of ATP at its original site similarly to erlotinib (Figure S5A,B). Different structural properties of EGFR ATP binding site in L858R–inhibitor complexes and binding energy of inhibitors and ATP in EGFR L858R–inhibitor–ATP complexes are summarized in Table 2, while details of hydrogen bonding, and all the other non-polar interactions are shown in Tables S1–S6. The results indicate that Wi-N and Wi-A, individually or in combination, could be effective against the L858R mutant.

In the case of exon19del EGFR, both Wi-A and Wi-N kept EGFR in an active state as observed in the case of erlotinib, and the distance between Lys745 and Glu762 had decreased due to binding of inhibitors (Table 2). Docking scores of the compounds, namely Wi-A (at Chain A −3.63 Kcal/mol; at Chain B −1.79 Kcal/mol) and Wi-N (at Chain A −4.28 Kcal/mol; at Chain B −1.52 Kcal/mol), were comparable to already known inhibitor erlotinib (at Chain A −5.28 Kcal/mol; at Chain B −3.20 Kcal/mol). Further, the DGF motif as well as αC-helix orientation was in inward direction in both exon19del–Wi-A and exon19del–Wi-N complexes (Table 2). Furthermore, similar to erlotinib, both Wi-A and Wi-N could inhibit the binding of the ATP at its binding site (Figure S5D,E). 

Further, the potential of Wi-A and Wi-N to act as wildtype EGFR protein inhibitor was explored. In the case of wildtype EGFR–Wi-N complex, the docking score was −3.99 Kcal/mol at Chain A, while at Chain B it was −6.66 Kcal/mol. Similarly, in the case of EGFR–Wi-A complex, docking score at Chain A was −5.744 Kcal/mol and at Chain B was −6.66 Kcal/mol. The orientation of the αC-helix and DFG motif was found to be inward in the case of both wildtype EGFR–Wi-A and wildtype EGFR–Wi-N complex. In the case of wildtype EGFR–Wi-N complex, the distance between Lys745 and Glu762 followed a similar trend as in the case of erlotinib and CAPE: it decreased in Chain A to 3.3 A and increased in Chain B to 7.8 A. In the case of wildtype EGFR–Wi-A complex, the distance between Lys745 and Glu 762 was reduced in both chains (Table 2). Further, ATP could not bind at its active site in wildtype EGFR in the presence of both Wi-A and Wi-N (Figure S5G,H).

Considering the ability of Wi-A to inhibit all exon 20 insertion mutants except D770_N771^InsNPG^ and that of Wi-N to inhibit the activity of all exon 20 insertion mutants except H773_V774^InsH^, it can be deciphered that the combination of Wi-A and Wi-N could inhibit the activity of all four exon 20 insertion mutants forms of EGFR studied here along with common L858R and exon19del mutations and wildtype EGFR.

### 3.8. CAPE Could Serve as Inhibitors for the Activity of Wildtype EGFR and Exon 20 Insertion Mutants Only

The interactions of CAPE at ATP binding site of wildtype EGFR and its common mutants L858R and exon19del were investigated and compared with that of interactions of erlotinib. Superimposition of wildtype EGFR–erlotinib complex with wildtype EGFR–ATP complex showed that the distance between Lys745 and Glu762 got reduced in Chain A from 6.4 to 4.7 Å while in Chain B it got increased from 6.5 A to 7.5 Å. However, the orientation of αC-helix and DFG motif was inward in both the chains, indicates that erlotinib maintains the active state of EGFR protein. In addition, erlotinib successfully inhibited the binding of ATP at its binding site in both the chains. In the case of wildtype EGFR–CAPE complex, the docking score of CAPE was higher than erlotinib, at Chain A it was −6.189 Kcal/mol and at Chain B −5.769 Kcal/mol. CAPE maintained the active conformation of wildtype EGFR protein by keeping αC-helix and DFG motif orientation in an inward direction. The distance between Lys745 and Glu762 decreased in Chain A from 6.4 to 3.9 Å but in Chain B it increased from 6.5 to 10 Å. At both chains, CAPE successfully inhibited the binding of ATP at its binding site in wildtype EGFR (Figure S5I). However, in the case of EGFR L858R and exon19del mutants, CAPE could not inhibit binding of ATP at its binding site (Figure S5C,F). All the above findings suggest that CAPE was only effective against wildtype EGFR and exon 20 insertion mutants except H773_V774^InsH^ but not effective for the common mutants of the EGFR.

## 4. Discussion

Lung cancer is the leading cause of cancer related deaths worldwide [1]. An Erb family kinase protein, EGFR, which plays a vital role in epithelial cell functions and growth [62]. However, its constitutive expression due to the amplification or mutation in its gene is the leading cause of the initiation and proliferation of NSCLC [12,63]. There are multiple synthetic drugs which are in advanced stage of clinical trials and some are approved as EGFR inhibitor such as gefitinib, erlotinib, poziotinib, osimertinib, lapatinib and many more [64,65,66,67]. In this study, erlotinib was taken as positive control for wildtype EGFR, exon 19 and exon 21 mutants while poziotinib and TAS6417 was taken as positive control against exon 20 insertion mutants of EGFR. Erlotinib has been approved for not only wildtype EGFR metastasis but also for mutated EGFR NSCLC. Moreover, it has been reported that erlotinib can increase the overall survival of patients with wildtype EGFR tumors [68,69]. A poziotinib phase II trial drug has shown a notable activity against exon 20 insertion mutation in *in vitro* and *in vivo* models. It should also be noted that poziotinib has shown a better activity than other approved inhibitor such as erlotinib and gefitinib in *in vitro* and xenograft models of exon 20 insertion mutants [13,21]. Further, TAS6417 has been shown to inhibit the exon 20 insertion mutation more significantly than the wildtype EGFR in *in vitro* and patient derived xenograft models [22,70]. However, the rapid increase of resistance of cancer cells toward the drugs and the adverse effects of the synthetic drugs on cancer patients has attracted researchers towards the natural drugs. Natural medicine such as Ashwagandha and honeybee propolis have been used for centuries for prevention and cure of multiple chronic and infectious diseases [41,71,72]. The anticancer activities of non-toxic dose of natural drugs such as Wi-A and Wi-N from Ashwagandha and CAPE from honeybee propolis have been reported through various mechanisms in the literature [34,36,73,74]. The selective inhibition of cancer cells has also been shown by the alcoholic leaf extract of Ashwagandha and Wi-N via ROS signaling [75]. In a recent *in vitro* study, it has been reported that a non-toxic dose of Wi-A alone could eliminate the drug-tolerant persistors, while in combination with phloretin it could effectively inhibited the growth of gefitinib resistant tumor in lung adenocarcinoma cell lines [38]. At the same time, Wi-N has been highly reported for its anticancer activities by inhibiting the survivin protein and abrogation of p53–mortalin complex and TPX2–Aurora complex [27,76,77]. While CAPE has mostly been found effective against breast cancer and nasopharyngeal carcinoma cells via targeting EGFR, it showed dose dependent inhibition of both the total and phosphorylated forms of EGFR protein expression (0–40 µM) [46]. The ADME/toxicity (Adsorption, Distribution, Metabolism and Excretion) prediction of Wi-A, Wi-N and CAPE through Qikprop tool of Schrodinger suite has been reported [78,79]. It has been predicted in previous studies that all three natural compounds, Wi-A, Wi-N and CAPE, have more than 80% of human oral absorption and they all follow the Lipinski rule of five [78,79,80], having moderate to no risk in terms of toxicity [79,81]. 

Although some studies have shown the possible inhibitory effect of the withanolides Wi-A and Wi-N and CAPE against the different forms of EGFR through cellular assays, there has been no study to the best of our knowledge showing the mechanism of the inhibition at the molecular level using these natural compounds [38,46]. Taking the reported anticancer activities of these biomolecules into account and the knowledge gap of their molecular mechanism of action against EGFR, in this study, we compared the potential of natural compounds Wi-A, Wi-N and CAPE to serve as ATP competitive inhibitors to already reported drugs. Here, we investigated the potentials and predicted the mechanism of action of the natural compounds towards aberrant EGFR mediated lung cancers through molecular docking, dynamics and free energy calculations. We found that Wi-A, Wi-N and CAPE could serve as potential ATP competitive inhibitors of wildtype and mutant forms of EGFR. All natural compounds bound at the same site and formed interactions with most of the common interacting residues of already reported drugs erlotinib, poziotinib and TAS6417. The same computational analysis was also performed with afatinib, which belongs to a class of covalently interacting and irreversible second-generation EGFR inhibitor, to get insight into the similarities of its interaction with our covalently interacting control inhibitors, poziotinib and TAS6417 and our natural compounds. It was found that, in all EGFR exon 20 insertion mutant–afatinib complexes, afatinib showed stable and covalent interaction at ATP binding site as reported in the literature and kept the protein in the active state by maintaining αC-helix-*in*, DFG-*in* and extended conformation of activation loop (Figures S6A–D and S7A–D). However, in the case of V769_D770^InsASV^ mutant of EGFR, a slight outward bend in αC-helix was observed, but it could not induce outward pivoting of the entire αC-helix from the pivot point and the ionic interaction between Lys745 and Glu762 was intact (Figure S6C). In addition, afatinib was able to compete with ATP very well when simulated together at the ATP binding site and completely displace ATP to another site in all EGFR exon 20 insertion mutant–afatinib–ATP complexes (Figure S6E–H). The interactions formed by afatinib at the ATP binding site were also found to be similar to those formed by natural substrate ATP, our control and test inhibitors (Tables S1–S6 and Figure S7). This brings us to the conclusion that the mechanism of action of natural compounds was similar to that of control compounds, and they all could serve as competitive inhibitors of EGFR mutants (with few exceptions) by locking the protein in active conformation but not letting ATP bind and carry out its function (Figure 3 and Figure 4 and Figures S4 and S5). Specifically, CAPE was more potent in the case of wildtype EGFR and exon 20 insertion mutants, as CAPE was binding at the ATP binding site with strong binding affinity and ATP was unable to displace it, but it could not serve as inhibitors for H773_V774^InsH^, L858R and exon19del mutants. It may also cause inhibition of phosphorylation of EGFR, keeping it in its inactive state. In the L858R mutants and exon19del, withanolides were efficient in inhibiting the binding of ATP and could not be displaced from their position when ATP was added, and thereby could inhibit the phosphorylation of EGFRs proteins keeping them in an inactive state. Moreover, it was observed that a combination of Wi-A and Wi-N may target the activity of all EGFR mutants studied along with that of wildtype EGFR protein. This *in silico* study suggests that Wi-A, Wi-N and CAPE may be recruited for the treatment of aberrant EGFR driven lung cancers. Although an extensive structure-based computational study was carried out including flexible docking, MD simulations and rescoring using MM/GBSA binding free energy calculations, there is a chance that the computationally calculated binding energies may not compare with actual affinity. The MM/GBSA methods have certain limitations such as use of implicit solvation model and ignorance of entropy changes during protein–ligand complex formation [82]. However, it has been reported that the lower is the MM/GBSA binding energy the stronger is the binding of ligand with the target, and this method can be useful in screening lead compounds [83]. Therefore, the ligand binding energies calculated in this study using MM/GBSA may not be the absolute binding energies but only the relative affinity of the ligands towards EGER with respect to each other. Hence, it is warranted that these molecules be tested *in vitro* and *in vivo* for any confident claim about their activities against EGFR and its mutants. 

## 5. Conclusions

In our study, molecular docking, classical MD simulations and MM/GBSA free energy calculations were used to investigate the binding specificity and dynamics of three natural compounds, Wi-A, Wi-N and CAPE, bound with the wildtype EGFR and its mutants. The overall analysis suggests that the combination of Wi-A and Wi-N could bind and stably interact at the binding site of the ATP in exon19del, L858R and exon 20 insertion mutants of EGFR. The interactions of CAPE with active site residues of ATP binding site were stronger and significantly more than Wi-A and Wi-N in the case of WT-EGFR and exon 20 insertion mutants. However, for common mutants of EGFR, L858R and exon19del, CAPE could not inhibit the binding of ATP at its site, hence could not serve as an inhibitor for these mutants. These tested natural molecules showed a decent binding affinity compared to control drugs, hence they may serve as readily available and safer alternatives for cancer therapy. However, these data require *in vitro* and *in vivo* experimental validation for the recruitment of these compounds for lung cancer treatment. This study may help in understanding the biological activity and mechanism of action of EGFR and its mutant. Further, it may contribute to rational drug design for novel targeted EGFR related cancer therapies.

## Figures and Tables

**Table 1:** Influence of compounds on different structural properties of EGFR exon 20 insertion mutants as ATP competitive inhibitors. The binding free energy is given in Kcal/mol and the distances are in Å.

EGFR Exon 20 Insertion Mutant		D770_N771^InsNPG^		D770_N771^InsSVD^		V769_D770^InsASV^		H773_V774^InsH^	
Inhibitor	Properties	Chain A	Chain B	Chain A	Chain B	Chain A	Chain B	Chain A	Chain B
Erlotinib	Binding Energy of inhibitor	−42.68	−41.12	−66.41	−64.28	−46.87	−61.40	−46.35	−45.27
	Binding Energy of ATP	−32.37	−24.83	−16.17	−27.34	−22.62	−27.44	−20.40	−25.51
	DFG motif and αC-helix orientation *	*In*	*In*	*In*	*In*	*In*	*In*	*In*	*In*
	Distance between Lys745 and Glu762	3.0 and 3.0	2.8 and 3.4	2.8 and 3.5	2.8 and 3.7	2.8 and 4.2	2.8 and 3.6	2.8 and 3.2	4.3 and 4.8
	Downward shift of P loop	No	No	No	yes	yes	yes	No	No
Poziotinib	Binding Energy of inhibitor	−59.55	−70.14	−48.17	−61.34	−69.83	−52.96	−60.82	−63.21
	Binding Energy of ATP	−20.56	−25.61	−22.40	−26.60	−33.42	−11.91	−53.86	−33.76
	DFG motif and αC-helix orientation *	*In*	*In*	*In*	*In*	*In*	*In*	*In*	*In*
	Distance between Lys745 and Glu762	3.1 and 3.0	3.3 and 3.0	2.9 and 5	2.9 and 3.7	3.5 and 5.6	2.9 and 3.1	2.8 and 4.6	2.8 and 3.3
	Downward shift of P loop	yes	yes	yes	No	No	No	No	No
TAS6417	Binding Energy of inhibitor	−39.31	−43.73	−71.92	−43.88	−54.74	−62.26	−47.06	−57.40
	Binding Energy of ATP	−34.17	−20.42	−32.55	−13.39	−36.68	−35.79	−16.83	−22.77
	DFG motif and αC-helix orientation *	*In*	*In*	*In*	*In*	*In*	*In*	*In*	*In*
	Distance between Lys745 and Glu762	2.8 and 3.6	2.8 and 4.2	6.4 and 8.3	3.0 and 3.0	2.8 and 3.9	2.9 and 3.2	4.5 and 6.0	2.9 and 3.3
	Downward shift of P loop	No	yes	No	yes	yes	yes	No	No
CAPE	Binding Energy of inhibitor	−56.48	−46.79	−70.04	−52.52	−63.94	−57.29	−44.17	−35.49
	Binding Energy of ATP	−28.92	−28.82	−20.88	−36.59	−29.20	−17.95	−39.10	−40.25
	DFG motif and αC-helix orientation *	*In* and *Out*	*In*	*In*	*In*	*In*	*In*	*In*	*In*
	Distance between Lys745 and Glu762	10.6 and 12.7	2.9 and 3.0	3.4 and 4.8	2.8 and 3.1	2.8 and 4.2	2.8 and 5.0	3.9 and 4.4	2.8 and 3.5
	Downward shift of P loop	No	yes	No	No	yes	yes	No	No
Wi-A	Binding Energy of inhibitor	−86.90	−46.23	−30.44	−25.60	−53.51	−45.11	−50.64	−42.62
	Binding Energy of ATP	−45.06	−30.59	−56.43	−62.40	−20.97	−38.84	−23.17	−35.66
	DFG motif and αC-helix orientation *	*In*	*In*	*In*	*In*	*In*	*In*	*In*	*In*
	Distance between Lys745 and Glu762	7.3 and 9.2	3.2 and 4.2	4.1 and 5.8	4.1 and 5.8	2.8 and 4.6	2.8 and 3.9	4.9 and 6.0	2.8 and 4.3
	Downward shift of P loop	No	No	No	No	yes	yes	No	No
Wi-N	Binding Energy of inhibitor	−48.28	−39.06	−62.79	−58.97	−38.08	−9.87	−35.58	−37.44
	Binding Energy of ATP	−20.17	−26.49	−26.82	−33.29	−60.96	−50.39	−13.47	−33.15
	DFG motif and αC-helix orientation *	*In*	*In*	*In*	*In*	*In*	*In*	*In*	*In*
	Distance between Lys745 and Glu762	5.6 and 7.5	2.8 and 3.5	2.8 and 3.1	7.8 and 9.5	2.8 and 4.7	3.2 and 5.0	6.0 and 8.1	2.9 and 3.0
	Downward shift of P loop	No	Yes	No	Yes	Yes	Yes	No	No

* ‘In’ refers to DFG and αC-helix inward conformation; ‘Out’ refers to DFG and αC-helix outward conformation.

**Table 2:** Activity of compounds on different structural properties of EGFR and its mutants as ATP competitive inhibitors. The binding free energy is given in Kcal/mol and the distances are in Å.

EGFR Mutants		exon19del		EGFR_L858R		Wildtype EGFR	
Inhibitor	Properties	Chain A	Chain B	Chain A	Chain B	Chain A	Chain B
Erlotinib	Binding Energy of inhibitor	−46.61	−36.29	−63.30	−56.41	−62.06	−40.37
	Binding Energy of ATP	−35.92	−32.26	−38.51	−37.26	−38.94	−56.57
	DFG motif and αC-helix orientation *	*Out*	*In*	*In*	*In*	*In*	*In*
	Distance between Lys745 and Glu762	3.6 and 3.1	3.4 and 3.2	3.9 and 2.8	9.6 and 6.4	6.4 and 4.7	6.5 and 7.5
	Downward movement of P loop	No	Yes	Yes	No	No	Yes
CAPE	Binding Energy of inhibitor	−38.15	− 58.83	−45.43	−49.22	−65.68	−63.23
	Binding Energy of ATP	−37.61	−34.63	−26.92	−23.62	−58.39	−30.15
	DFG motif and αC-helix orientation *	*In*	*In*	*In*	*In*	*In*	*In*
	Distance between Lys745 and Glu762	3.6 and 3.5	3.4 and 3.7	3.9 and 3.1	9.6 and 6.5	6.4 and 3.9	6.5 and 10.1
	Downward movement of P loop	Yes	yes	Yes	Yes	No	Yes
Wi-A	Binding Energy of inhibitor	−49.35	−46.23	−22.52	−44.50	−49.50	−48.78
	Binding Energy of ATP	−45.06	−30.59	−56.43	−62.40	−35.78	−35.78
	DFG motif and αC-helix orientation *	*In*	*In*	*In*	*In*	*In*	*In*
	Distance between Lys745 and Glu762	3.6 and 3.4	3.4 and 3.2	3.9 and 3.1	9.6 and 6.4	6.4 and 3.5	6.5 and 6.4
	Downward movement of P loop	No	No	No	No	No	Yes
Wi-N	Binding Energy of inhibitor	−59.83	−22.21	−45.76	−51.86	−44.88	−51.70
	Binding Energy of ATP	−14.15	−23.45	−20.82	−20.29	−35.42	−37.25
	DFG motif and αC-helix orientation *	*In*	*In*	*In*	*In*	*In*	*In*
	Distance between Lys745 and Glu762	3.6 and 3.6	3.4 and 3.2	3.9 and 3.7	9.6 and 6.4	6.4 and 3.3	6.5 and 7.8
	Downward movement of P loop	Yes	Yes	Yes	Yes	No	No

* ‘In’ refers to DFG and αC-helix inward conformation; ‘Out’ refers to DFG and αC-helix outward conformation.

## Data Availability

The authors confirm that the data supporting the findings of this study are available within the article and/or its supplementary materials.

## Supplementary Materials

The following are available online at https://www.mdpi.com/2218-273X/11/2/160/s1, Figure S1: (**A**) Protein and ligand RMSD plot of 500 ns simulation trajectory of EGFR D770_N771^InsSVD^–poziotinib complex; and (**B**) superimposition of average representative structure of EGFR D770_N771^InsSVD^–poziotinib complexes obtained from stable trajectories of 50 ns simulation (yellow-red) and 500 ns simulation (grey-blue), Figure S2: (**A**) Protein RMSD plots of simulation trajectories of different EGFR exon 20 insertion mutant proteins. Protein RMSD plots for different EGFR exon 20 insertion mutant–ligand complexes: (**B**) EGFR_D770_N771^InsNPG^; (**C**) EGFR_D770_N771^InsSVD^; (**D**) EGFR_V769_D770^InsASV^; and (**E**) EGFR_H773_V774^InsH^ mutants, Figure S3: Superimposition of active and inactive conformation of EGFR–ATP complex, Figure S4: Interaction of control and test compounds at the ATP binding site of Chain A of EGFR exon 20 insertion mutant–inhibitor complexes. Superimposition of wildtype EGFR–ATP (grey-blue) complex with EGFR exon 20 insertion mutant–inhibitor (yellow-red) complexes of: D770_N771^InsNPG^ (**A**–**F**); D770_N771^InsSVD^ (**G**–**L**); V769_D770^InsASV^ (**M**–**R**); and H773_V774^InsH^ (**S**–**X**) mutants of EGFR. P Loop and activation segment of wildtype EGFR–ATP complex is shown in marine blue and purple color and that of EGFR exon 20 insertion mutant–poziotinib complex is shown in salmon and firebrick red color, Figure S5: Superimposition of wildtype EGFR–ATP (grey-blue) complex with EGFR L858R–inhibitor–ATP complexes (yellow-red-pink): (**A**) EGFR L858R–Wi-A–ATP complex; (**B**) EGFR L858R–Wi-N–ATP complex; and (**C**) EGFR L858R–CAPE–ATP complex. Superimposition of wildtype EGFR–ATP (grey-blue) complex with EGFR exon19del–inhibitor–ATP complexes (yellow-red-pink): (**D**) EGFR exon19del–Wi-A–ATP complex; (**E**) EGFR exon19del–Wi-N–ATP complex; and (**F**) EGFR exon19del–CAPE–ATP complex. Superimposition of wildtype EGFR–ATP (grey-blue) complex with EGFR wildtype–inhibitor–ATP complexes (yellow-red-pink): (**G**) EGFR wildtype–Wi-A–ATP complex; (**H**) EGFR wildtype–Wi-N–ATP complex; and (**I**) EGFR wildtype–CAPE–ATP complex. P Loop and activation segment of wildtype EGFR–ATP complex is shown in marine blue and purple color and that of EGFR wildtype–inhibitor–ATP complexes and EGFR mutant–inhibitor–ATP complexes is shown in salmon and firebrick red color, Figure S6: Interaction of afatinib at ATP binding site of EGFR exon 20 insertion mutant–afatinib and EGFR exon 20 insertion mutant–afatinib–ATP complexes. Superimposition of wildtype EGFR–ATP (grey-blue) complex with EGFR mutant–afatinib (yellow-red) complexes (**A**–**D**) and EGFR mutant–afatinib–ATP (yellow-red-pink) complexes (**E**–**H**) with EGFR exon 20 insertion mutants: D770_N771^InsNPG^ (**A**,**E**); D770_N771^InsSVD^ (**B**,**F**); V769_D770^InsASV^ (**C**,**G**); and H773_V774^InsH^ (**D**,**H**). P Loop and activation segment of wildtype EGFR–ATP complex is shown in marine blue and purple color and that of EGFR exon 20 insertion mutant–poziotinib complex is shown in salmon and firebrick red color, Figure S7: Interactions formed by afatinib at ATP binding site of: (**A**) D770_N771^InsNPG^; (**B**) D770_N771^InsSVD^; (**C**) V769_D770^InsASV^; and (**D**) H773_V774^InsH^ mutants of EGFR. Interactions formed with key ATP interacting residues are highlighted in purple circles, Table S1: EGFR mutants interactions with erlotinib in presence and absence of ATP, Table S2: EGFR mutants interactions with poziotinib in presence and absence of ATP, Table S3: EGFR mutants interactions with TAS6417 in presence and absence of ATP, Table S4: EGFR mutants interactions with CAPE in presence and absence of ATP, Table S5: EGFR mutants interactions with Wi-A in presence and absence of ATP, Table S6: EGFR mutants interactions with Wi-N in presence and absence of ATP.
