# Supplementary material for: Computational Insights into the Potential of Withaferin-A, Withanone and Caffeic Acid Phenethyl Ester for Treatment of Aberrant-EGFR Driven Lung Cancers
Source: Biomolecules. 2021 Jan 26;11(2):160. doi: 10.3390/biom11020160 (PMC7911128; doi:10.3390/biom11020160)

Supplementary Figure 1

A

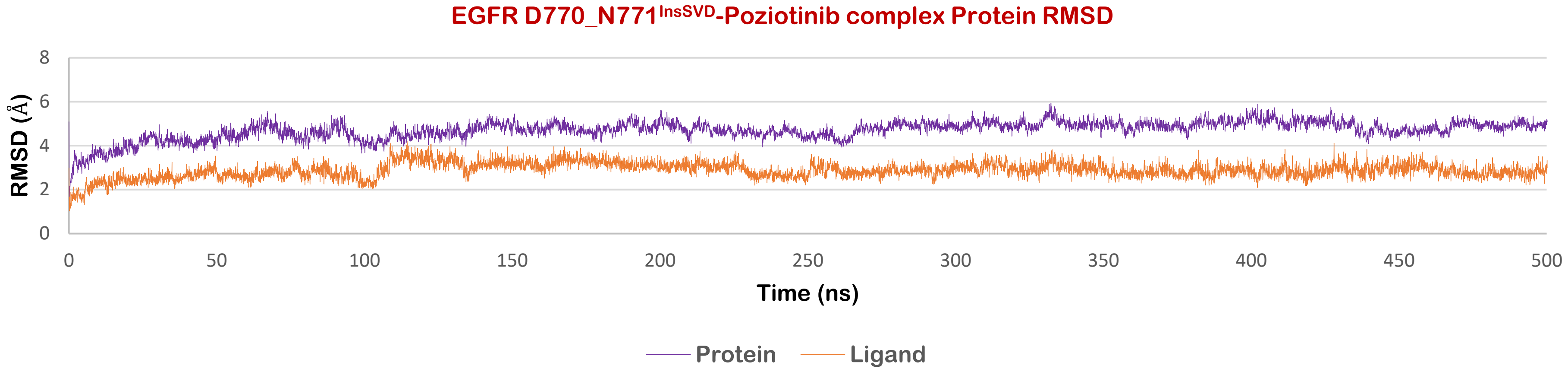

B

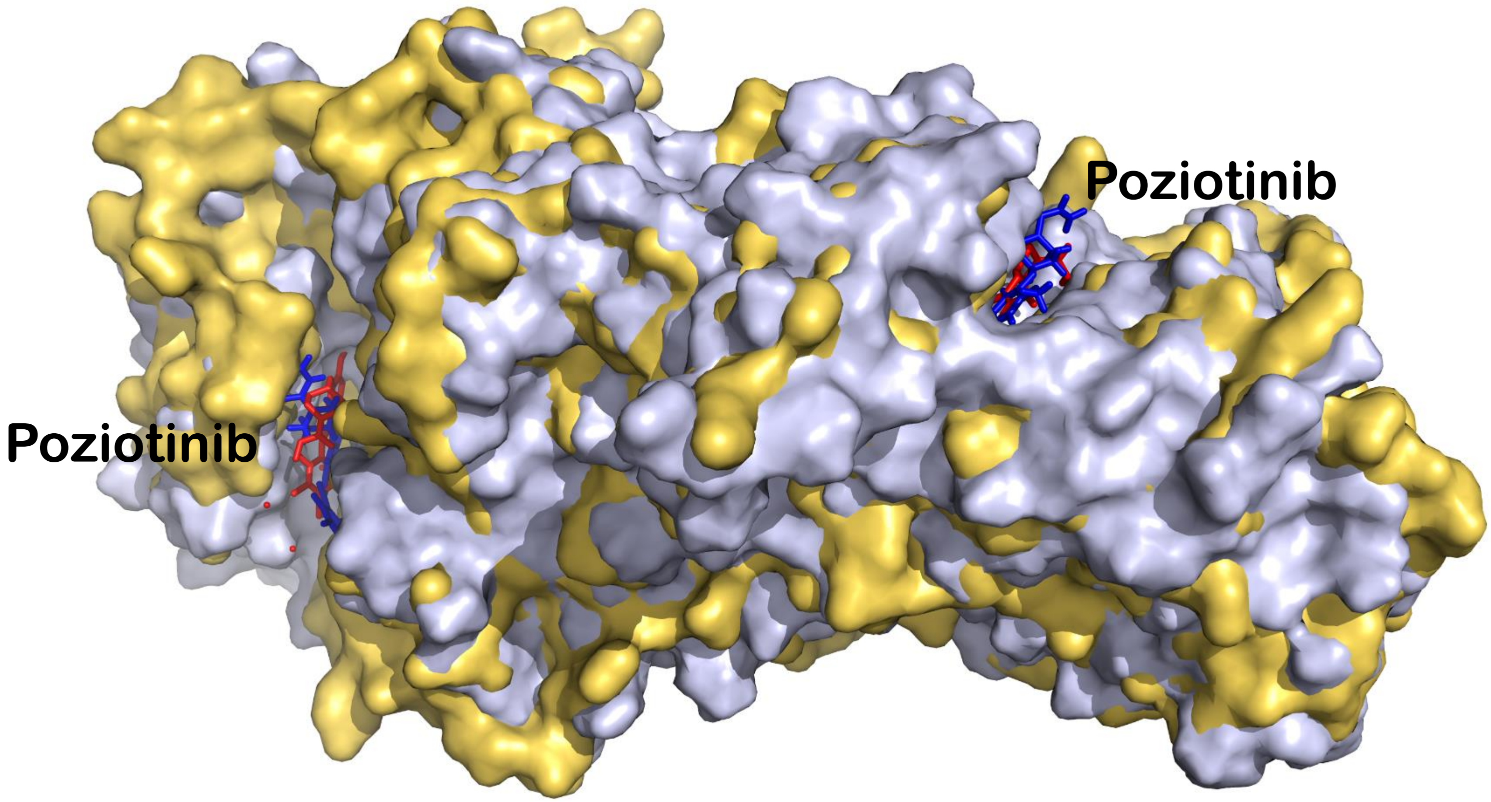

Supplementary Figure 2

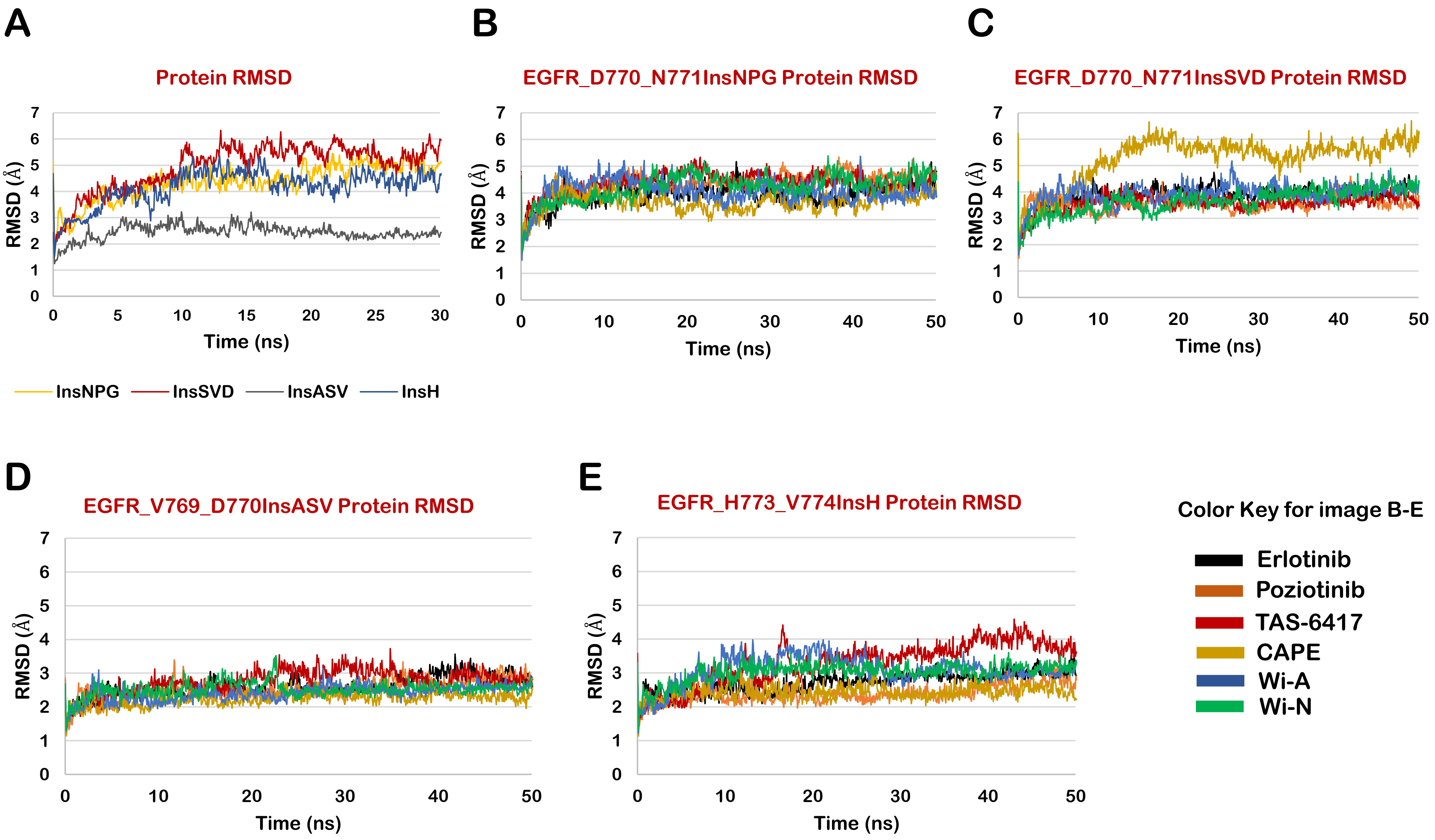

Supplementary Figure 3

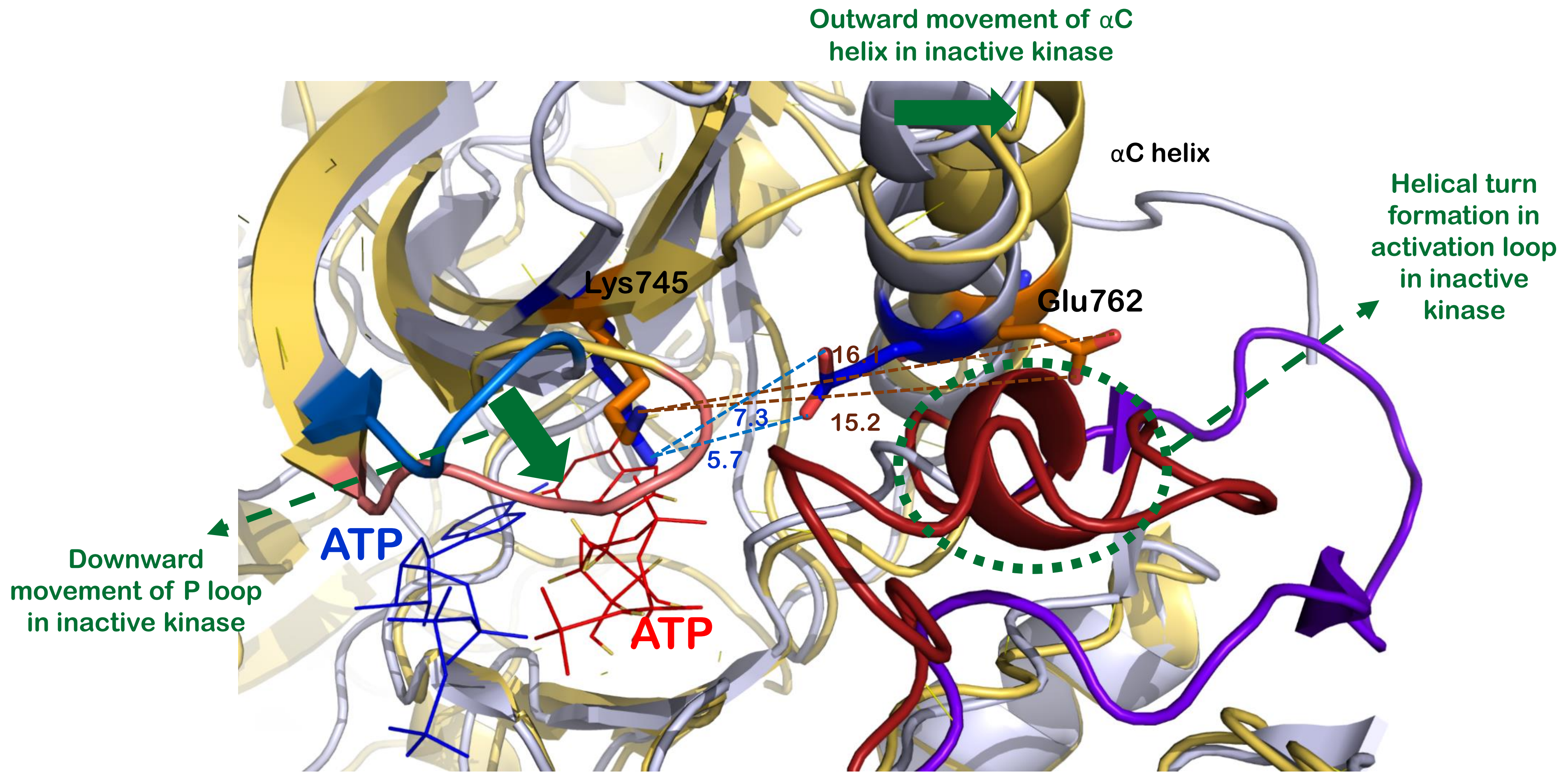

EGFR-ATP Active Kinase
P Loop
Activation Loop

EGFR-ATP Inactive Kinase
P Loop
Activation Loop

Supplementary Figure 5

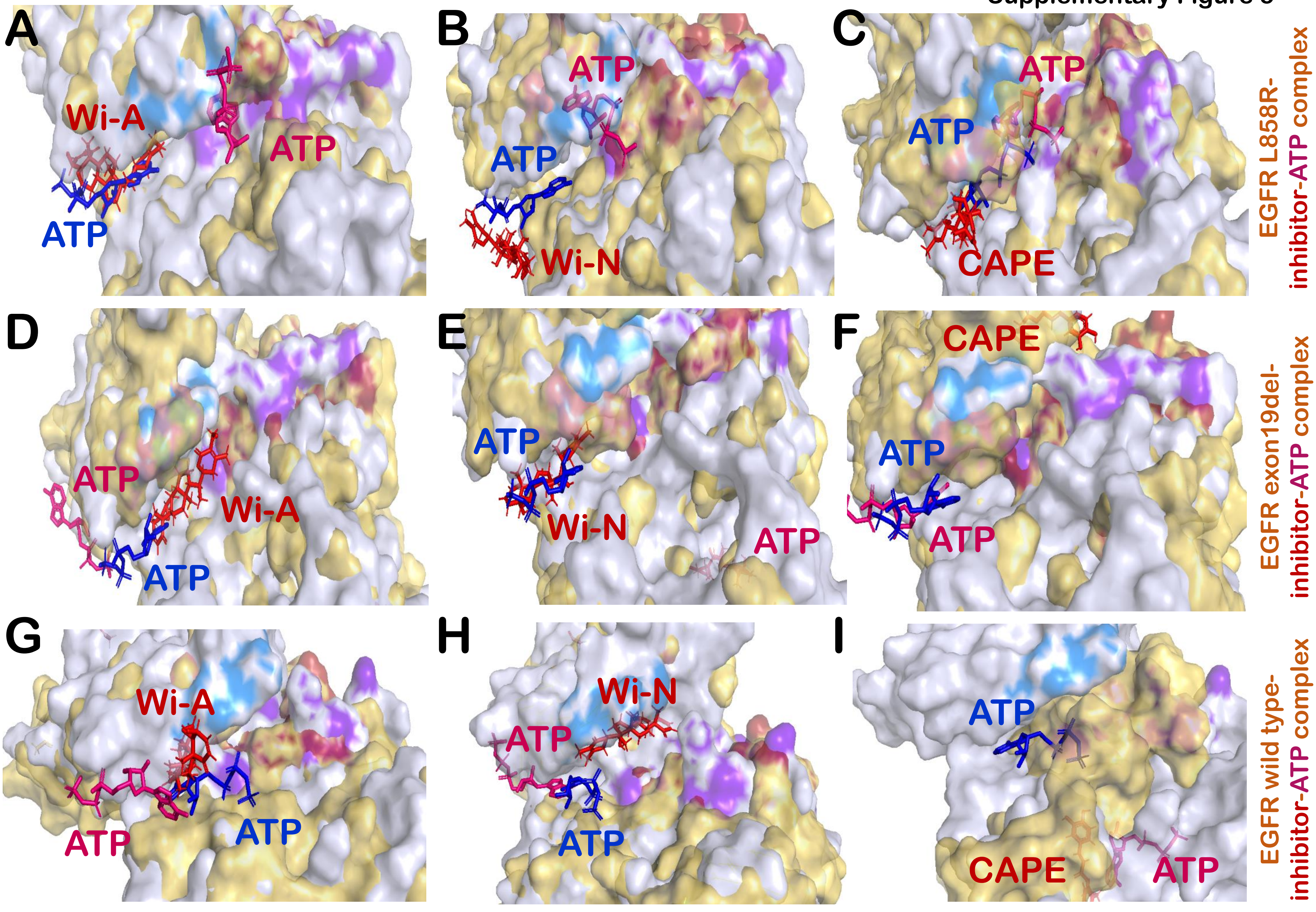

Supplement: Supplementary file 1 [file biomolecules-11-00160-s001.zip › 20200828-Suppl-Figures-1-3and5.pdf]
