# Supplementary figures and images for: Computational Insights into the Potential of Withaferin-A, Withanone and Caffeic Acid Phenethyl Ester for Treatment of Aberrant-EGFR Driven Lung Cancers
Source: Biomolecules. 2021 Jan 26;11(2):160. doi: 10.3390/biom11020160 (PMC7911128; doi:10.3390/biom11020160)

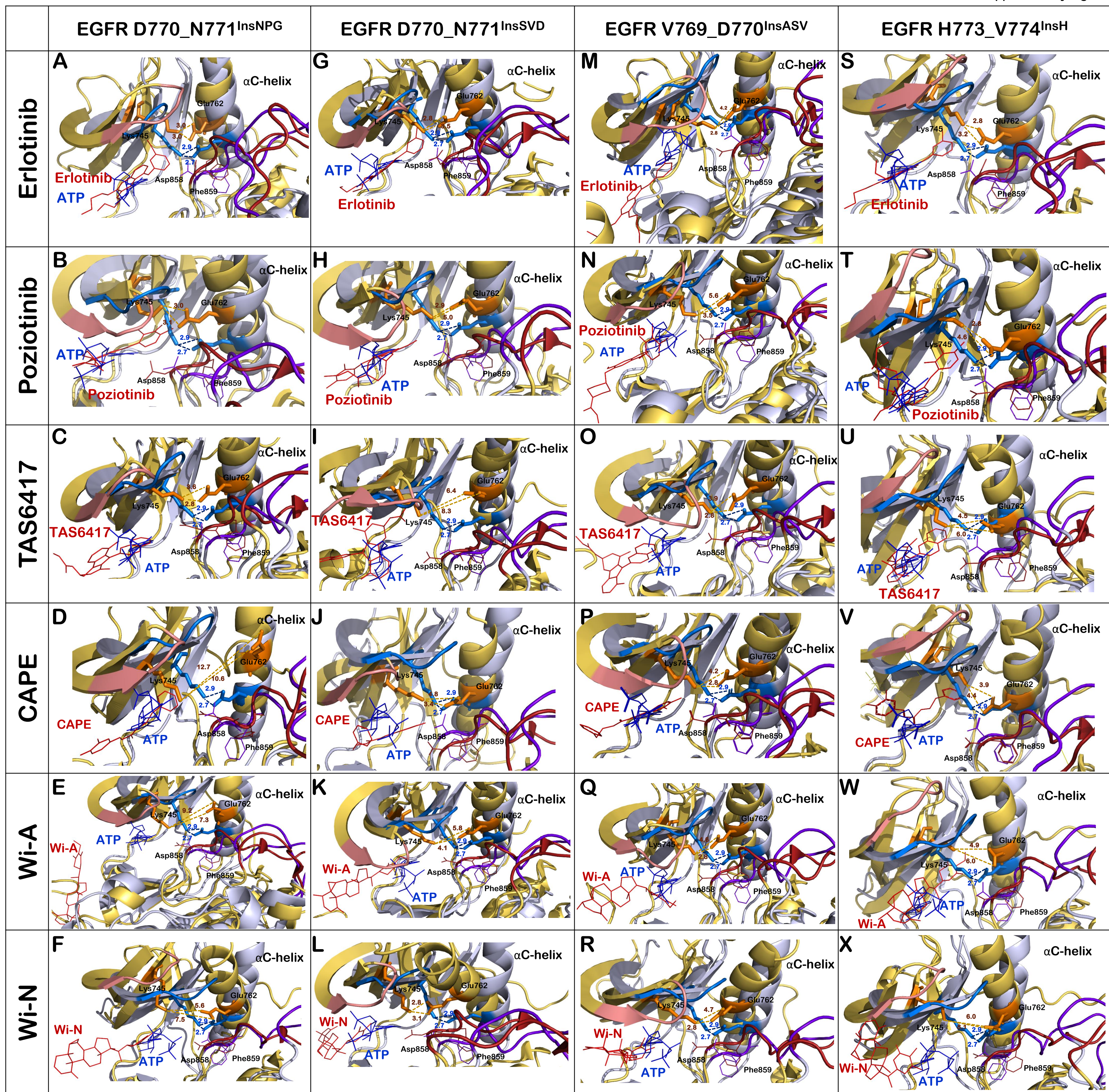

Supplement: Supplementary file 1 [file biomolecules-11-00160-s001.zip › Supplementary Fig4.pdf]

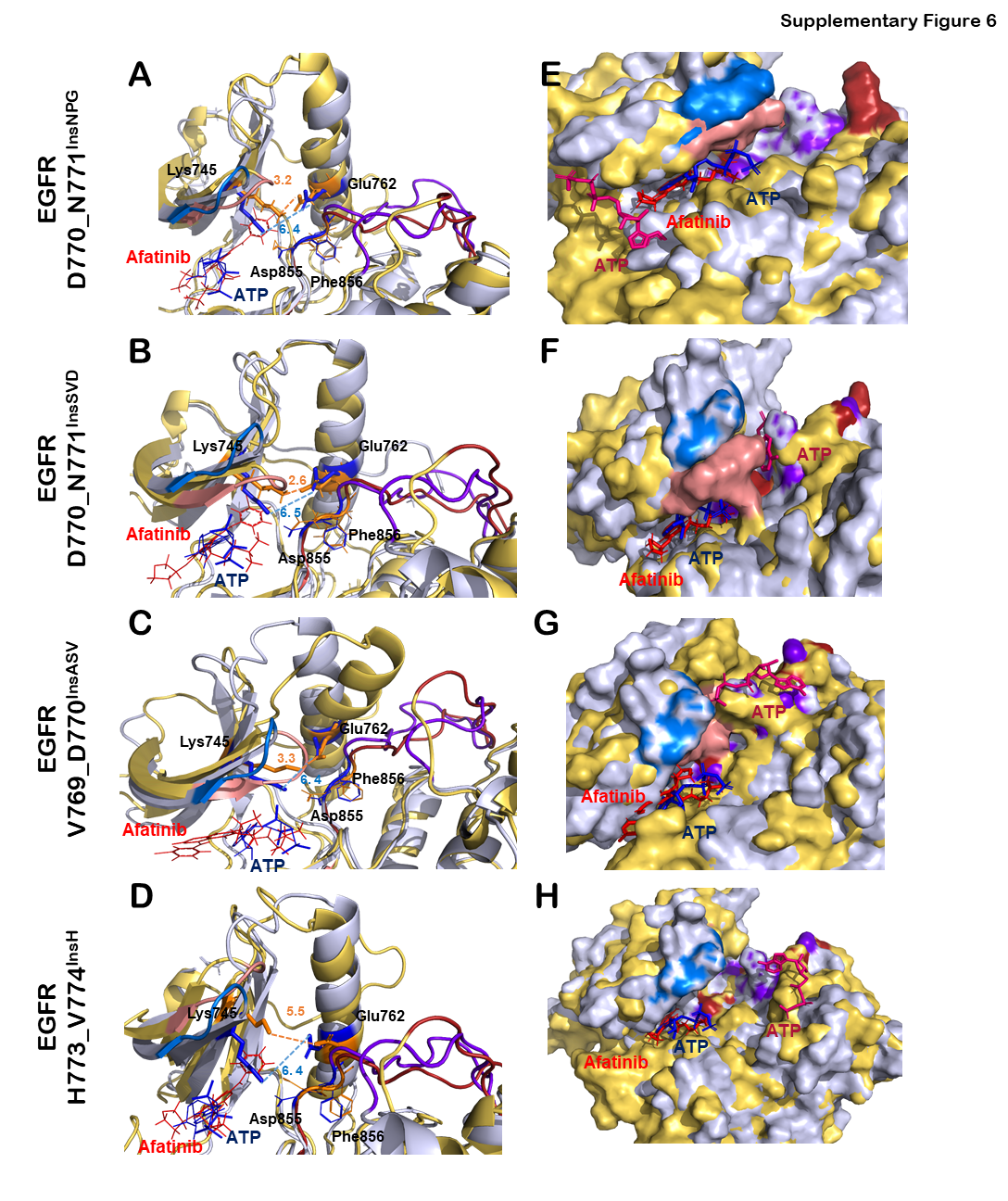

Supplement: Supplementary file 1 [file biomolecules-11-00160-s001.zip › Supplementary Fig6.TIF]

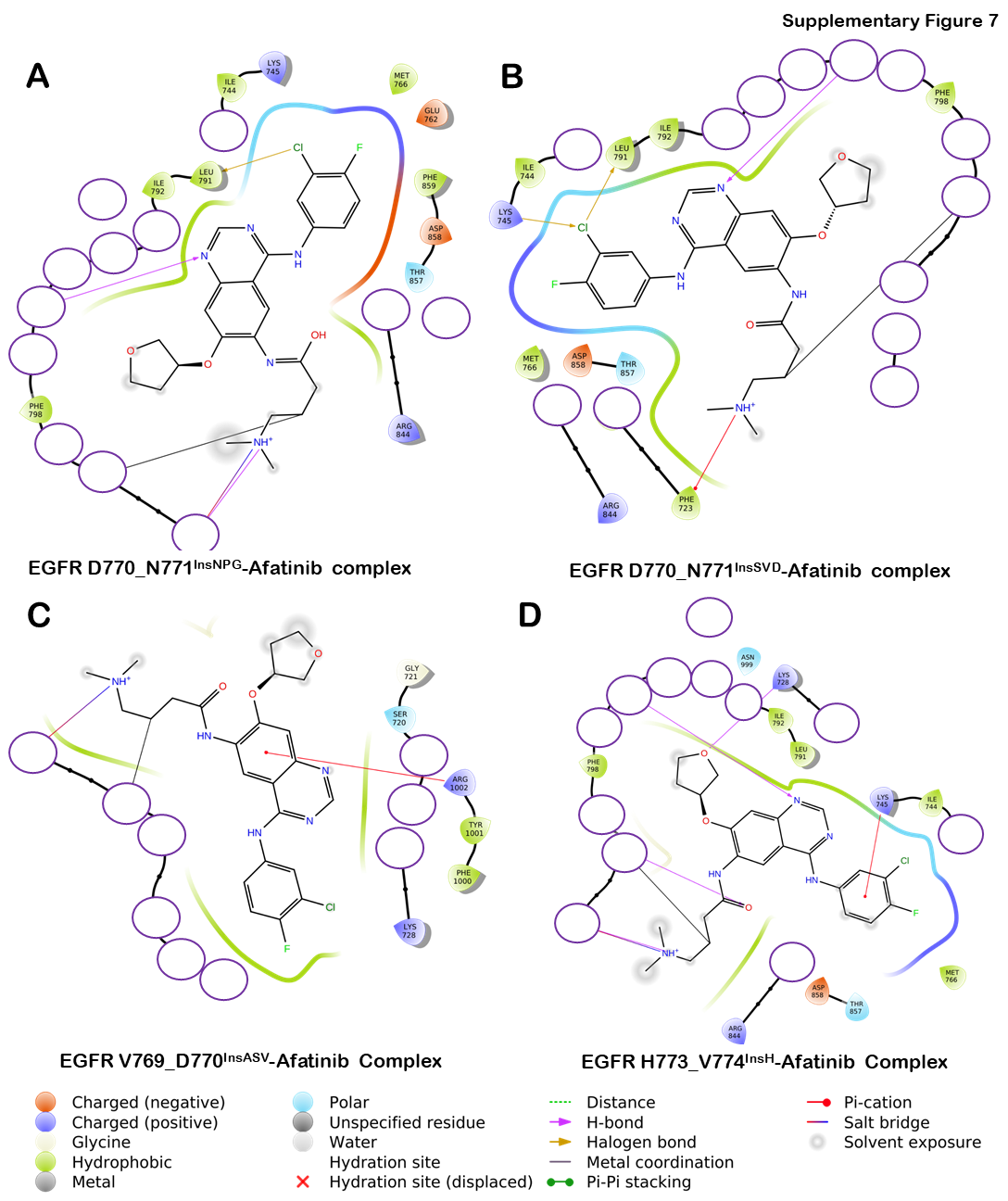

Supplement: Supplementary file 1 [file biomolecules-11-00160-s001.zip › Supplementary Fig7.TIF]
